# Influence of transgenesis on genome variability in cucumber lines with a *thaumatin II* gene

**DOI:** 10.1007/s12298-021-00990-8

**Published:** 2021-04-28

**Authors:** Agnieszka Skarzyńska, Magdalena Pawełkowicz, Wojciech Pląder

**Affiliations:** Department of Plant Genetics, Breeding and Biotechnology, Institute of Biology, Warsaw University of Life Sciences, Warsaw, Poland

**Keywords:** Comparative genomics, Cucumber, Small nucleotide polymorphism, SNP, Transgenesis, Thaumatin

## Abstract

The development of new plant varieties by genetic modification aims at improving their features or introducing new qualities. However, concerns about the unintended effects of transgenes and negative environmental impact of genetically modified plants are an obstacle for the use of these plants in crops. To analyze the impact of transgenesis on plant genomes, we analyze three cucumber transgenic lines with an introduced *thaumatin II* gene. After genomes sequencing, we analyzed the transgene insertion site and performed variant prediction. As a result, we obtained similar number of variants for all analyzed lines (average of 4307 polymorphisms), with high abundance in one region of chromosome 4. According to SnpEff analysis, the presence of genomic variants generally does not influence the genome functionality, as less than 2% of polymorphisms have high impact. Moreover, analysis indicates that these changes were more likely induced by in vitro culture than by the transgenesis itself. The insertion site analysis shows that the region of transgene integration could cause changes in gene expression, by gene disruption or loss of promoter region continuity.

**Supplementary Information:**

The online version contains supplementary material available at 10.1007/s12298-021-00990-8.

## Introduction

The development of genetic modification methods has created the possibility of developing plants with an increased or decreased expression of given genes or with transgenes introduced from other species. The creation of genetically modified (GM) plants aim at improving or introducing new features in crop plants; for obtaining better quality, resistance to stress factors, and higher yields; and thus better use in agriculture and horticulture. However, genetic modifications and the use of GM plants raise concerns about the unintended impact of a transgene on modified plants, the spread of the transgene, and the negative environmental impact of such crops. Therefore, the introduction of GM plants for cultivation involves the accurate determination of possible environmental risks, as well as the safe use of such varieties for human and animal nutrition.

Concerns about obtaining and using GM plants are most often associated with the occurrence of unintended transformation effects, when in addition to the intended change from the introduction of a foreign gene, there are additional changes in the genome sequence. The appearance of polymorphisms in the genome sequence can affect the structure and expression of genes, and thus changes in the functioning of proteins and disorders of metabolic pathways in the entire organisms. While larger changes in genomes, such as larger insertions, deletions, and translocations, can cause phenotypic changes, significant dysfunction in plant organisms, or even be lethal, small variants up to several nucleotides are often not observed at the phenotypic level. However, changing a single nucleotide can also cause very serious disorders, examples of which are the many human genetic diseases (Yue and Moult 2006; Moszyńska et al. 2017).

The development of next generation sequencing (NGS) methods has complemented the methods of molecular analysis of GM plants used to date, such as Southern blotting to analyze the number of transgene copies or fluorescence in-situ hybridization to determine the integration site. The use of re-sequencing allows, at relatively low cost, the determination of the location of transgene integration, the way of insert incorporation, the number of copies, and the changes in the genome that insertion of the transgene sequence brings (Yang et al. 2013). Although NGS allows for faster and more efficient analysis, there is a concern of usage of this method stand-alone in a GM plants risk assessment (Pauwels et al. 2015). Yet such quickly developing technology could soon be sufficient for complete analysis of transgenic plants.

Our experiments aim to show whether transgenesis and transgene integration at a random location in the genome induce genome-wide changes and if these changes influence the genomic structure and integrity. For this purpose, we used three transgenic cucumber lines with an introduced gene for thaumatin II (Szwacka et al. 2000). Earlier analysis carried out on these lines determined the gene copy number and chromosomal location (Szwacka et al. 2000, 2002; Tagashira et al. 2005) but did not show its position in the cucumber genome. Metabolic and organoleptic analyses (Szwacka et al. 2002, 2012; Tagashira et al. 2005), and lastly profiles of mRNA and miRNA (Pawełkowicz et al. 2020), were also performed, but no comparative genomic analysis was made to evaluate changes throughout the genome. Therefore, in this study, we performed re-sequencing and comparative genomic analyses to determine changes in the genomes of transgenic lines relative to the B10 cucumber reference line used for transformation. Resequencing and typing of polymorphisms also allowed identification of genome sites that are more likely to change.

## Materials and methods

### Plant material, cultivation, and DNA isolation

Transgenic lines with introduced *thaumatin II* gene were derived from the highly inbred homozygous cucumber B10 line. We chose lines from three independent transformation events: 212, 224, and 225, obtained by *Agrobacterium tumefaciens* transformation as it was described in Szwacka et al. (2002); the lines were self-pollinated and for this analysis, the T9 generation was used. Transgenic plants were cultivated during the 2014 summer season in a field experiment. DNA was extracted from 100 mg of young leaves (frozen and ground in liquid nitrogen) with the DNeasy Plant Mini Kit (Qiagen, Germany) according to manufacturer’s protocol. The yield and quality of the DNA isolation was checked by electrophoresis on a 1% agarose gel and spectrophotometrically with NanoDrop2000 (Thermo Fisher, USA).

### Sequencing, read pre-processing, and mapping

Sequencing of the DNA was done using an Illumina HiSeq2000 system with 100-bp-long paired-end reads. The raw sequencing data were deposited in the NCBI Sequence Read Archive under BioProject (PRJNA638559). The sequencing reads underwent quality analysis with FastQC and removal of leftover Illumina adapters, low-quality bases (sliding window of 4 bases, average Phred score of 30), and fragments shorter than 50 bases using Trimmomatic ver. 0.35 (Bolger et al. 2014). Afterwards, pre-processed sequencing reads were corrected with the BFC tool ver. r181 (Li 2015). Each read set was mapped separately to the B10v3 reference genome (GenBank: LKUO00000000) (Osipowski et al. 2020) with BowTie2 v.2.2.9 software (Langmead and Salzberg 2012), and output alignments were deduplicated with Samblaster ver. 0.1.24 tool (Faust and Hall 2014).

### Identification of transgene insertion sites

The insertion sites of transgenes were detected based on the method presented by Park et al. (2017). Illumina sequencing reads were mapped to the reference genome B10v3 (Osipowski et al. 2020) and also to the vector sequence used for the transformation: pRUR528 with *thaumatin II* cDNA under 35SCaMV promoter and *nptII* gene under the nopaline synthase promoter (Szwacka et al. 2002). Results were visualized with the IGV tool (Robinson et al. 2017). Reads mapped to the plasmid and their paired reads were used to indicate the insertion sites in the genomes of cucumber transgenic lines. To confirm the transgene integration sites genomes of transgenic lines were assembled de novo from Illumina reads with BowTie2. The insertion sites were confirmed by PCR analysis with primers specific to these junction sites. The list of primers used for validation is shown in Table S1.

### Variant prediction and comparative genomics

For variant calling we used two software packages: (1) Freebayes ver. 1.1.0-3-g961e5f3 and (2) DeepVariant ver. 0.4.1 using the independent algorithms, described by Osipowski et al. (2020). Each line was analyzed separately. To increase the variant prediction accuracy results of Freebayes, variant calling was subjected to a series of filters according to Li (2014): low-complexity region exclusion, quality filter of minimum variant quality = 30 (Phred), unbiased double-strand coverage, minimum read depth > 10, and maximum read depth < 29 on average. For detection of repetitive sequences used in low-complexity filtering, the Mdust script was used. The DeepVariant results that passed both of the two built-in quality filters were cross-checked with the results of Freebayes. The final data consists of single-nucleotide variations (SNVs) common to both methods. Comparisons of variant data files were performed with BCFtools ver. 1.4–6-g5349659 and BEDtools v2.26.0 (Quinlan 2014). In the analysis of polymorphism distribution within each genome, we used a chromosome map that was prepared based on contig mapping using cucumber markers, as described in Skarzyńska et al. (2020). The histograms representing the density of variants were created with the circlize package for R, version 0.4.8 (Gu et al. 2014). Polymorphisms were counted within bins 10 000 nt long.

### Verification of predicted polymorphisms

The variant calling method was experimentally verified by PCR amplification and Sanger sequencing for 30 randomly selected polymorphisms. Primers were designed to flank the variant region, based on the reference genome B10v3 sequence. We excluded polymorphisms predicted in homopolymeric regions from the analysis. The used primers are listed in the supplementary material (Table S2). Sequencing reads were then analyzed, and if the polymorphic region differed from the reference sequence (as in the variant call file), the result was considered as positive. If the region was similar to the reference, the verification result was considered as negative.

### Localization of SNVs and effect prediction

Localization of predicted variants was done with BCFtools ver. 1.4–6-g5349659, by comparison with the reference genome annotation. Variants were assigned to the following genomic regions: genes (exons, introns, and untranslated regions [UTRs]), gene upstream and downstream regions (both 1500 bp in length), and intergenic regions. Estimation of the effect of the predicted variants on genes and encoded proteins were performed with the SnpEff ver 4.3 T program (Cingolani et al. 2012) using a cucumber database built with the reference genome B10v3 and its annotations (Osipowski et al. 2020). The prediction of SNV effects were done with settings for searching only canonical transcripts, and the length of upstream and downstream regions were set as 1500 bp (1500 bp before the gene start codon and after the gene stop codon).

### KOG and gene ontology analysis

Genes in which polymorphisms had a strong influence (high-impact [HI] variants) according to SnpEff analysis and genes located on ctg1556 were classified into eukaryotic orthologous groups (KOGs) using the KOGnitor database available at NCBI.

## Results

### Sequencing data

Sequencing of cucumber transgenic lines with the *thaumatin II* gene (hereafter called thaumatin lines) resulted in 126.6–126.8 million paired-end reads for each of the three analyzed lines (Table S3). The sequencing coverage was 35 × for all analyzed samples. Mapping to the reference B10v3 genome (Osipowski et al. 2020) used high-quality reads, which represented 97% of all reads. Of these reads 84–89% were mapped successfully, of which 95–96% mapped uniquely to the reference genome. These uniquely mapped reads were used for further analysis.

### Identification of transgene insertion sites

**Fig. 1:**
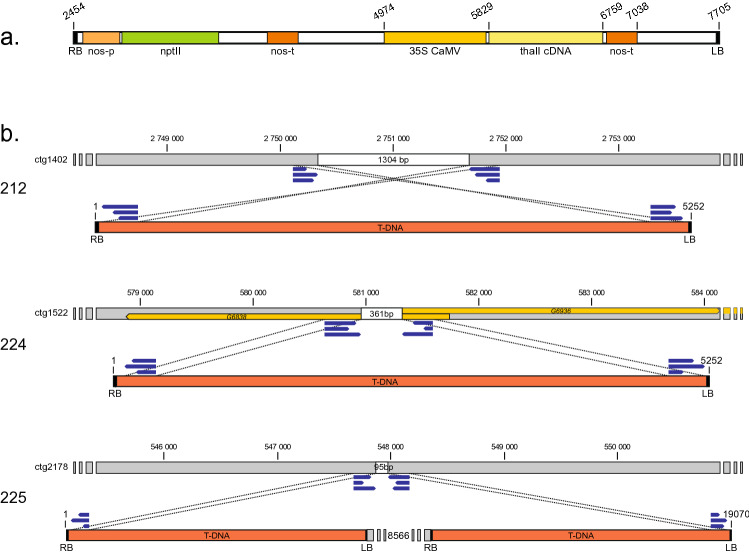
**a** Scheme of the T-DNA used for transformation. **b** The exact sites in specific contigs, and the way of integration of the inserted T-DNA. The deletion within the insertion site is marked with a white bar; dotted lines mark the junction sites indicated by chimeric read pairs mapped to the reference genome on one side and to the T-DNA sequence on the other side

To confirm the presence of transgene sequences and identify the insertion sites, paired-end reads were mapped to the reference genome and to the plasmid sequence used for transformation. Reads were successfully mapped to the transgene in all analyzed lines, and mapping results indicated that the site and the way of transgene integration were different for each line (Table 1, Fig. 1A). In 212 line, the transgene is located at chromosome 6, ctg1402. In 224 line, it is integrated at chromosome 2, ctg1522, whereas in 225 line, it is also located at chromosome 2 but at ctg2178. Mapping results showed that in 212 and 224 lines, the T-DNA region is integrated into the genome sequence as one copy. In 225 line, the two copies of the T-DNA with whole vector sequence are integrated (Fig. 1B). These results were confirmed by PCR analysis with primers specific to the junction sites. Mapping analysis indicated rearrangement occurring during transgene insertion caused deletions in the DNA strand: from 95 nt in 225 line, 361 nt in 224 line, and 1304 nt in 212 line. Further study of the integration sites shows that in 212 and 225 lines, the transgene is located in the intergenic region. In 224 line, insertion is within the promoter of the *G6936* gene, directly preceding the transcription start site (− 1 bp from TSS) and disrupting the *G6838* gene located at the reverse strand (with deletion of the first 142 bp of the coding sequence).

**Table 1:** Information about thaumatin II transgene insertion site in the cucumber genome

Line	Junction site	Deletion in the insertion site	Transgene insertion	Integration site
212	Chr 6; ctg1402	1304 nt	T-DNA region; 1 copy	Intergenic region
224	Chr 2; ctg1522	361 nt	T-DNA region; 1 copy	Promoter of *G6936* gene, disruption of *G6838* gene
225	Chr 2; ctg2178	95 nt	T-DNA—vector backbone—T-DNA; 2 copies	Intergenic region

### Variant calling

Genomic variants between analyzed thaumatin lines and the reference genome were identified by using two tools, Freebayes and DeepVariant, which are based on independent algorithms. Comparison of the results from both two variant calling algorithms gave highly reliable predictions (Table 2). The number of predicted SNVs was similar among the three lines. Freebayes predicted 4652, 3975, and 4426 SNVs, whereas DeepVariant gave 80 246, 79 076, and 81 108 variants for 212, 224, and 225 lines, respectively. Comparison of the two variant calling methods gave the final number of polymorphisms, which is 4610, 3929, and 4383 for 212, 224, and 225 lines, respectively. These polymorphisms were used in subsequent analysis.

**Table 2:** Number of SNVs predicted by Freebayes and DeepVariant, and after cross-checking. Variant rate is the number of variants per 1b of genomic sequence

	Freebayes	DeepVariant	Common	Variation rate
212	4652	80,246	4610	1.35 × 10^−5^
224	3975	79,076	3929	1.15 × 10^−5^
225	4426	81,108	4383	1.28 × 10^−5^
Mean	4351	80,143	4307	1.26 × 10^−5^

**Fig. 2:**
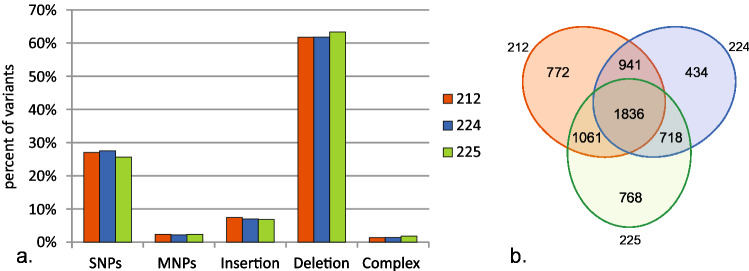
**a** Number of particular types of predicted variants in cucumber thaumatin lines. **b** Venn diagram of common polymorphisms in transgenic lines

All lines showed similarity in the percentages of particular types of variants, such as single nucleotide polymorphisms (SNPs), multi-nucleotide polymorphisms (MNPs), insertions, deletions, and complex changes (variants of mixed type). Most of the variants were deletions, which represented 62% of all changes. The second group was SNPs and MNPs, representing 27% and 2% of predicted polymorphisms, respectively, while insertions accounted for 7%. The complex changes represented less than 2% of variants (Fig. 2A).

### Comparison between lines

Comparison of predicted polymorphism within the three analyzed lines revealed that 1836 variants are common to all lines (40%, 47%, and 42% of predicted variants in 212, 224, and 225 lines, respectively). These variants comprise 427 SNPs, 47 MNPs, 101 insertions, 1243 deletions, and 18 complex variants (Fig. 2B). These common polymorphisms, mostly indels, are within homopolymeric regions. Line-specific variants accounted for 772 (17% of all predicted variants) in 212 line, 434 (11%) in 224 line, and 768 (18%) in 225 line.

### Variant distribution

**Fig. 3:**
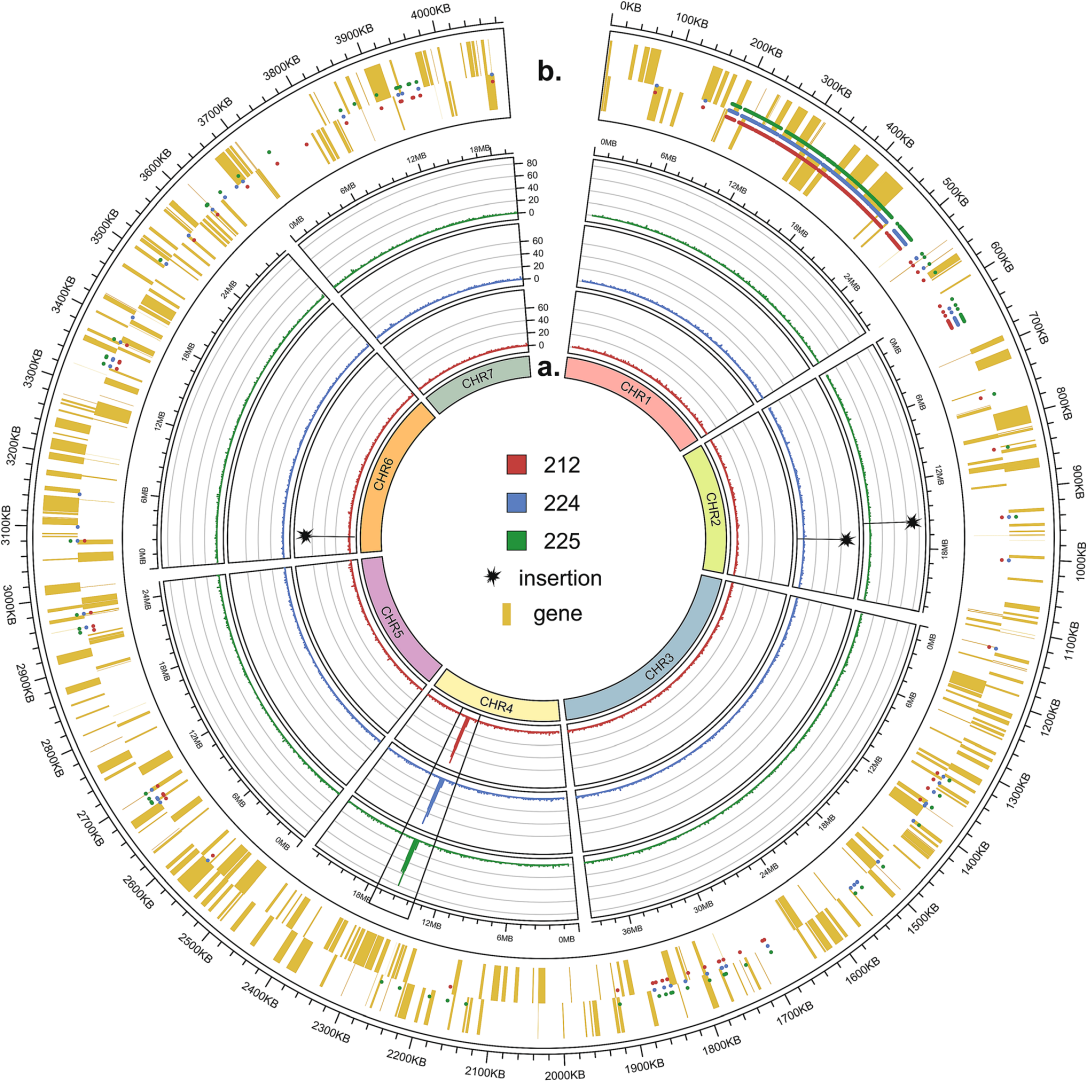
**a** Distribution of predicted variants on the cucumber chromosomes. Histograms show the number of polymorphisms within regions of 10 000 nt. The region on chromosome 4 with the highest density of polymorphisms is marked with a box. The transgene insertion sites are marked with asterisks. **b** Distribution of predicted polymorphism within ctg1556, which possesses the highest number of variants. Positions of variants are marked with dots, and yellow bars indicate genes (protein coding, lincRNA, tRNA, rRNA, snRNA, and miRNA) on plus and minus strands

Analysis of the variant localization on specific cucumber chromosomes was done by mapping of polymorphisms. This analysis showed a similar distribution among all thaumatin lines: variants were distributed equally within chromosomes with the exception of chromosome 4, where a high percentage of polymorphisms was located around the 15–16 Mb region (39%, 40%, and 38% in 212, 224, and 225 lines, respectively), mostly within ctg1556 (Fig. 3A). There is no correlation between the length of the chromosome and the number of predicted variants.

The genomic density of polymorphisms was calculated based on the total length of the reference genome B10v3, which is 342.3 Mbp. The density of SNPs and MNPs, insertions and deletions in the genome was similar for all three lines (Table S4). The highest density within the genome represented deletions (eight deletions per 1 Mb), followed SNPs and MNPs (four variants per 1 Mb). Insertions were the least common – only one insertion per 1 Mb.

### Description of predicted polymorphisms

The pattern of transitions (Ts) and transversions (Tv) was similar among all analyzed lines. Transitions (C/T and G/A changes) were 1.6–1.8 times more frequent than transversions (C/G, A/T, C/A, G/T). The most abundant changes were G/A transitions (31–33% of all SNPs) and C/T substitutions (30–32%). Among transversions, the most frequent changes were A/T and T/G substitutions (10–11%), followed by C/A (9–10%) and C/G changes (6–8%).

Deletions were about 8–9 times more frequent than insertions in all analyzed lines. Analysis of the length of indels showed that most changes were 1 bp in length (97–98% of deletions and 88–89% of insertions).

### Annotation of variants

Predicted variants were annotated to localize them within particular genomic regions and determine their most probable effect on genes and proteins. The density of changes annotated within exons, introns, UTRs, 1500-bp regions upstream and downstream of the gene sequence, and intergenic regions were similar for all analyzed thaumatin lines. The density of polymorphisms accounting for 1 Mb of particular regions was lowest in the exons (10–12 variants per 1 Mb) and highest in the intergenic regions (31–36 var/Mb) (Table S5). The density of variants in the UTRs, introns, and upstream and downstream regions was at a similar level (14–16 var/Mb, 17–20 var/Mb, and 15–18 var/Mb, respectively).

The effects of predicted polymorphisms on genes and encoded proteins were estimated with SnpEff, and the severity level was used to divide the results into four categories: high (assumed disruptive effect), moderate (non-disruptive effect, but still capable of influencing protein functionality), low (mostly harmless effect), and modifier (within non-coding regions or non-coding genes). By far, most of the predicted variants were classified as having a modifier effect (97–98%), which is in line with the localization of the majority of changes within non-coding genomic regions (Table S5, Table S6). The proportion of variants presenting moderate and low effects was less than 1%, whereas variants predicted as possessing high influence (impact) on the genes and proteins represented 1.9%, 1.8%, and 1.6% in 212, 224, and 225 lines, respectively. These HI variants were further analyzed.

Analysis of variants with SnpEff also allowed us to estimate the genome regions affected by predicted polymorphisms. Most of the variants had an effect on the intergenic sites of the genome (49–52%). Upstream and downstream regions were affected by 10–11% and 9% of all predicted variants, respectively. The majority of changes annotated within genic regions affected UTRs (21–24% in both 5′ and 3′ UTRs), followed by introns (18–20%). A smaller share of polymorphisms had an effect on exons (3–3.5%).

Polymorphisms located in gene regions were analyzed to determine whether they cause non-synonymous (NS) or synonymous (S) changes. In 224 and 225 lines, the NS/S ratio was 1.5, as the share of missense polymorphisms was 60%, while that of silent changes was 40%. In 212 line, the NS/S ratio was similar, 1.6, and the majority were NS variants (61%, including 58% missense SNPs).

### Genes containing variants with predicted high impact

Further analysis of variants with high impact on genes and encoded proteins revealed that 105, 89, and 85 genes were affected in 212, 224, and 225 lines, respectively (Table S7). All of the genes were protein coding. In total, 131 unique protein accessions were subjected to functional analysis, as the same genes were affected in more than one line.

The highest percentage of HI polymorphisms (95–97%) was represented by frameshift variants – insertions and deletions of a number of bases that was not divisible by 3. In 212 and 224 lines, variants causing loss of a start or stop codon represented around 2% polymorphisms, while those causing a gain of a stop codon represented 1% of HI variants. In 225 line, these variants were represented by 1% of HI polymorphisms (Table 3).

**Table 3:** Types of HI variants according to SnpEff analysis

	212		224		225	
frameshift_variant	108	95.58%	92	94.85%	87	96.67%
start_lost	2	1.77%	2	2.06%	1	1.11%
stop_gained	1	0.88%	1	1.03%	1	1.11%
stop_lost	2	1.77%	2	2.06%	1	1.11%

To investigate the functions of genes with HI variants, KOG analysis was performed (Figure S1). Except for genes of unknown function, it is hard to indicate significantly enriched groups. However, genes involved in post-translational modifications, signal transduction mechanisms, carbohydrate transport and metabolism, and transcription are more abundant in all analyzed lines.

### Experimental verification

Experimental verification was done by PCR amplification of regions containing polymorphisms followed by sequencing of the products. Thirty randomly chosen (excluding variants located at polynucleotide sites because the problems with primer design) SNVs were analyzed (10 per line): 16 SNPs, 5 MNPs, 7 insertions, and 2 deletions. The products obtained in 28 reactions were then sequenced, and the sequences of 25 variants were positively verified. Variant calling precision was estimated as 89%, which indicates high reliability of the performed polymorphism prediction.

### Analysis of ctg1556

Polymorphism distribution analysis showed that a high percentage of variants (on average 30% of all variants predicted for the thaumatin lines) were located within ctg1556 on chromosome 4. Most of the variants located in ctg1556 are SNPs and MNPs (82–84%). Deletions represent 9%, insertions 3–5%, and complex changes 4% of all variants. Distribution of polymorphisms within ctg1556 is not equal, as most of the variants are located at the beginning of the contig, from 200 to 500 kb (Fig. 3B). Many variants (516 polymorphisms, or 37–42% of all variants predicted) are similar for all three transgenic lines. Of the variants mapped in ctg1556, 23% are mainly in genic regions, and 86% of them are located in introns.

**Fig. 4:**
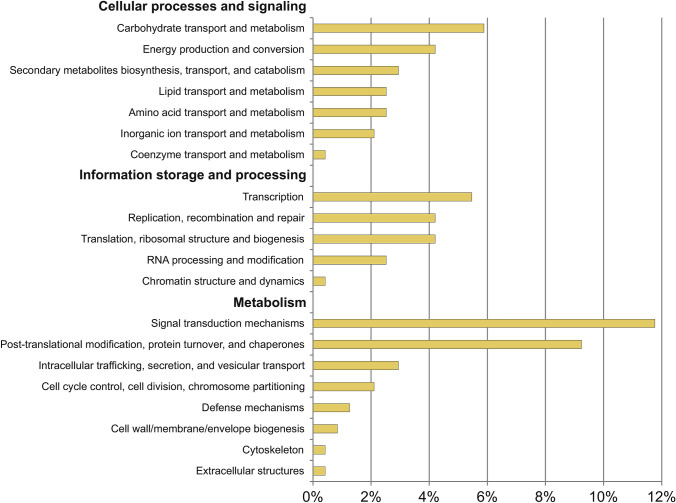
Functional KOG classification of genes localized on ctg1556

According to annotation, 363 genes are located in ctg1556 (both strands accounted together), of which 64% are coding proteins and 28% are lincRNA. KOG analysis of genes located on this contig indicates that most of them are engaged in cellular processes and signaling, mainly in signal transduction mechanisms and post-translational modification, protein turnover, and chaperones (Fig. 4).

Polymorphisms were present in 35 genes in 212 line, 34 genes in 224 line, and 29 genes in 225 line (Table S8). However, most of these are intron variants with a modifier impact on genes. Among the three lines, 10 genes (all of them protein coding) can be pointed out as having 20 or more variants within their structure: *G7757, G7760, G7761, G7762, G7763, G7764, G7765, G7949, G7950,* and *G7951.* According to KOG analysis, most of them are engaged in translation, ribosomal structure, and biogenesis.

## Discussion

NGS allows determination of transgene integration sites, and it provides a more detailed analysis of these sites compared to other methods used for this purpose, such as Southern blotting, segregation, or cytogenetic analysis. NGS also provides information on the whole genome sequence, which allows for study of the effect of transformation on the entire plant genome. In our study, we used NGS to analyze the T-DNA integration site and changes in the genome of transgenic cucumber lines, which can be induced by transformation. For this purpose, we re-sequenced genomes of three transgenic cucumber lines with an introduced *thaumatin II* gene. Genomes were compared to the reference genome of the B10 line, to predict genomic changes arising from transgenesis and to evaluate the risk of this method for obtaining new cultivars.

Mapping of sequencing reads to the reference genome showed that each of the transgenic lines has the transgene integrated into a different genomic site: for 212, 224, and 225 lines, the ctg1402, ctg1522, and ctg2178, respectively. In 212 and 224 lines, T-DNA is incorporated as one copy, while 225 line has a double copy of transgene together with the vector backbone. Despite the differences in the insertion site, the lines show a similar number of genome changes (SNPs, MNPs, and indels) as well as similar distribution of these changes, with the highest density of polymorphisms on chromosome 4, especially within ctg1556. At the transgene insertion site, deletions of different lengths were observed: 95 nt, 361 nt, and 1304 nt in 225, 224, and 212 lines, respectively. This is in agreement with earlier reports of deletions at the integration site (Salomon and Puchta 1998; Kleinboelting et al. 2015).

According to previous studies of transgene insertion sites, it was suggested that T-DNA incorporates into gene-rich and transcriptionally active sites of genomes, that is, within genes and promoter regions (Koncz et al. 1989; Chen et al. 2003). However, most of the previous analyses were carried out on selected lines showing transgene expression that was associated with a selection error (Gelvin 2017). Kim and Veena (2007) conducted research on the transgene insertion site without selection pressure based on transgene expression. It has been shown that the transgene insertion site is more random, without preferred sites. Thaumatin lines that were used in this study were regenerated in the selected conditions, and in two lines, 212 and 225, the transgene was integrated into the non-coding, intergenic regions, while in 224 line, the transgene was inserted into the promoter region of *G6936*, disrupting continuity of the *G6838* gene on the “minus” strand. Taking into account both described statements, our results could more support the hypothesis of Gelvin (2017) that T-DNA integration is rarely a clean event and, moreover, the phenomenon of T-DNA integration is often accompanied by other phenomena, such as deletions, insertions, or integration of binary vector backbone sequences.

Earlier studies on transgene copy number in thaumatin lines by segregation analysis and Southern blotting have indicated that 212 and 224 lines have a single copy of the transgene, while 225 line has two copies of the transgene (Szwacka et al. 2002; Yin et al. 2004). This was also confirmed in this study by NGS and read mapping to the reference genome and to the sequence of the vector used for transformation. The analysis of the transgene integration site also confirmed earlier cytogenetic analyses that indicated integration of the transgene at a single locus and the presence of the *thaumatin II* transgene on chromosome 2 in 225 line (near the centromere region) and on chromosome 6 in 212 line (interstitial region) (Tagashira et al. 2005). It should be noted that detection of a double transgene copy in 225 line by this method was not possible due to the type of insertion (Fig. 1B) and relatively low resolution.

During regeneration of transgenic lines with an introduced *thaumatin II* gene, no somaclonal variability was observed (Szwacka et al. 2000). The profile of the change distribution observed in transgenic lines, with a large proportion of variants found in chromosome 4 at the 15–16 Mb region, is similar to the results previously obtained for somaclonal lines (Skarzyńska et al. 2020). The somaclonal line described as S2 was obtained by regeneration of leaf explants (leaf callus regeneration) on a solid medium, as transgenic lines were obtained. The S2 somaclonal line presented a significant proportion of variants in the same region of chromosome 4 as the thaumatin lines, especially on ctg1556, within which 23% of all selected polymorphisms are located. Therefore, we suggest that the changes observed in the genomes of transgenic lines are mainly caused by regeneration in in vitro cultures, not the transformation and transgene insertion alone. Analyses carried out on transgenic soy (Anderson et al. 2016) and rice (Endo et al. 2015; Kawakatsu et al. 2013) also showed that tissue cultures, which are the stage used to obtain GM plants, induce more changes than transgenesis alone. Kawakatsu et al. (2013), in their comparative genomics analysis of rice, showed that much fewer variants occur between the transgenic line and the initial variety from which it was obtained, than between the initial variety and the variety from which it was derived. Moreover, they showed the genomic integrity between the initial and transgenic line, determined on the basis of RNA-seq analysis (Kawakatsu et al. 2013). The very process of obtaining transgenic plants is stressful to them due to preparation and cutting of explants, inoculation and co-culture, and selection conditions. All of these factors could induce genomic changes. As common variants in both the cucumber transgenic and somaclonal lines occur mostly within ctg1556, it is possible that this genomic region is a buffer site for this type of change. The ontology analysis showed that most of genes located at ctg1556 are engaged in signal transduction, post-translational modification, protein turnover, and chaperones.

Despite having passed through in vitro cultures, cucumber transgenic lines show a lower degree of change (average variation rate, calculated as number of variants per genome length, of 1.28 × 10^−5^) than that of cucumber somaclonal lines obtained as a result of regeneration of leaf explants on solid media (average variation rate of 2.35 × 10^−5^) (Skarzyńska et al. 2020). The background line for the transformation was the highly homozygous B10 line, which shows a very low spontaneous mutation ratio, as it was presented in Osipowski et al. (2020). In this study, different generations of B10 plant were compared. The average number of variants was 31, and average variation rate was 9.25 × 10^−8^. Kawakatsu et al. (2013) showed that the transformation mutation rate per week of culture is similar to the somaclonal mutation rate per week of culture (0.68 × 10^−7^ and 0.85 × 10^−7^, respectively). Results obtained for regenerated Arabidopsis showed that the mutation rate in in vitro propagated plants increased between 60 × and 350 × (Jiang et al. 2011). Similar results were obtained for rice, where the base change ratio in regenerants was 248 × higher than the spontaneous mutation rate in Arabidopsis (Miyao et al. 2012). In conclusion, our results, and also previous studies, showed that transgenesis causes relatively fewer changes than the breeding method by regeneration in in vitro cultures and most genomic changes in transgenic plants are caused rather by conditions and time explant spent in in vitro cultures than by the transformation itself.

Analysis of the expression level of the *thaumatin II* gene in cucumber transgenic lines showed that transgene expression occurs in all three thaumatin lines, but the level of expression varies (Pawełkowicz et al. 2020). The lowest expression level was obtained for 225 line, despite it having a double copy of T-DNA. This could be correlated with the mRNA threshold level, or it could be associated with the integration site being close to the centromeric region (Tagashira et al. 2005), which is transcriptionally less active. In the remaining lines, expression level of the *thaumatin II* gene is similar, with no noticeable effect of the transgene integration site, whether it is in an intergenic region (212 line) or inside the gene structure (224 line).

Regarding RNA-seq and miRNA-seq analyses in the fruits of three transgenic lines (212, 224, and 225), it was concluded that transformation of the cucumber genome with *thaumatin II* and expression of the transgene had minimal impact on gene expression and epigenetic regulation by miRNA in the cucumber fruits (Pawełkowicz et al. 2020). The next important step was a comparative genome analysis, which aimed to check if and how transgene insertion affects gene expression. As a result of the analysis of the insertion sites, it can be seen that in both lines, the transgene was integrated in an intergenic region at a distance from the nearest gene of 15 454 nt and 5326 nt in 212 and 225 lines. In 224 line, the integration site was in the genic region, where two genes overlapped on opposite DNA strands, *G6936* (strand “ + ”) and *G6838* (strand “-”). Previously, we revealed that these genes have altered expression: *G6936* is upregulated and *G6838* is downregulated in cucumber fruits. The detailed analysis showed that the insertion site was in a promoter region (− 1 bp from TSS) of *G6936,* and a higher level of expression could be connected with a strong constitutive promoter (35S CaMV) close to the TSS and could cause higher expression of *G6936.* The other explanation of upregulation of *G6936* is that, due to rearrangements, the interaction between *cis-*acting elements and transcription factors might influence the expression level. The transgene insertion could increase expression of *G6936.* The continuity of the *G6838* gene was broken by the transgene and the first 142 nt of the coding sequence was deleted, which could cause changes in the gene expression.

Despite relatively few variants predicted for thaumatin lines, most of them do not cause significant functional changes. Only 1% of polymorphisms selected in cucumber transgenic lines with the *thaumatin II* gene are expected to have a high impact on the formation and functioning of proteins. Variants located in the non-coding regions (intergenic regions, promoters, and exons) may influence the genes and proteins functions as well, but they have rather low or modifier impact, without significant changes to phenotype or plant physiology. No direct correlation was observed between the location of the variant in the genes and their variable expression in the sets of identified differentially expressed genes (DEGs) (Pawełkowicz et al. 2020). Only one gene located on ctg1556, *G7760*, was found to have altered expression in 224 and 225 lines compared with the control. The upregulated *G7760* encoded a protein that contained the pentatricopeptide repeat motif that has a role in mitochondria or plastids (Pawełkowicz et al. 2020). This could support the hypothesis discussed above, that changes observed in the genomes of transgenic lines are likely caused by the in vitro cultures, due to specific conditions that influence organelles. No significant correlation was observed between the polymorphism in this gene *(G7760)* and its variable expression. It seems that these are co-existing events that do not influence each other.

Schnell et al. (2015) suggested that if transgenesis and insertion causes a change profile in plant genomes similar to that caused by conventional plant breeding methods, they should pose a similar level of risk. In addition, no differences in genomic stability or the formation of new protein functions between GM and non-GM crops have been demonstrated (Weber et al. 2012). Our results support this conclusion, because in comparison to genomes of cucumber somaclones lines (coming from the same B10 background) (Skarzyńska et al. 2020) the changes noticed herein are less abundant, indicating that they were likely spontaneous. Moreover, the number of differentially expressed genes in fruit (which is a market product) of transgenic lines is relatively small, indicating scant influence on the genomic stability (Pawełkowicz et al. 2020).

## Conclusions

We conclude that transgenesis of cucumber plants induces relatively low genomic variation, which is most likely the result of the in vitro culture stage of the transformation procedure, rather than transgene integration itself. However, the insertion site of the transgene could have an impact on the genomic features localized at that site. It could change the gene expression due to gene disruption or modification of regulatory regions. The genomic variants in transgenic lines are distributed rather equally, but around one-third of all polymorphisms are clustered on a 2-Mb-long region of chromosome 4 (ctg1556), the same as in the somaclonal lines (Skarzyńska et al. 2020) generated by the same method as described for transgenic lines. This can indicate that this region is more susceptible to change under the influence of stress factors that occur during in vitro culture. The comparative genomic results of non-GM and GM cucumber lines, and analysis of differentially expressed genes between these lines (Pawełkowicz et al. 2020), supports the postulate that they should show a similar level of risk.

## Supplementary Information

Below is the link to the electronic supplementary material.

Supplementary file1 (DOCX 271 KB)

## Data Availability

Sequence data that support the findings of this study have been deposited in the Sequence Read Archive (SRA) with the BioProject accession code PRJNA638559.
