# Supplementary material for: Influence of transgenesis on genome variability in cucumber lines with a thaumatin II gene
Source: Physiol Mol Biol Plants. 2021 Apr 28;27(5):985–96. doi: 10.1007/s12298-021-00990-8 (PMC8139995; doi:10.1007/s12298-021-00990-8)
Supplement: Supplementary file 1 — Supplementary file1 (DOCX 271 KB) [file 12298_2021_990_MOESM1_ESM.docx]

**Supplementary information**

**Table S1**. The list of primers used in the junction sites analysis. *Names in brackets indicate expected product sizes for particular analyzed line.

| **Name** | **Sequence** | **Expected product size [bp]*** |
| --- | --- | --- |
| **Primers specific for vector (confirming presence of vector backbone)** | | |
| Tha_B_F | CTCTTCGTTCGTCTGGAAGG | 219 |
| Tha_B_R | CGGGCAGGATAGGTGAAGTA |  |
| Tha_A_F | GGAGTGAAAGAGCCTGATGC | 230 |
| Tha_A_R | GCCGGTATAAAGGGACCACC |  |
| Tha_A_F | GGAGTGAAAGAGCCTGATGC | 3306 (B10); 6934 (212);  7909 (224); 8190 (225) |
| Tha_B_R | CGGGCAGGATAGGTGAAGTA |  |
| **Primers specific for insertion site in 212 line** | | |
| 1FL_F | TTGTAGGACGGTGTTGACGG | 2988 (B10) 6616 (212) |
| 1FL_R | TCCCCGACCCCAACTCTTTA |  |
| Tha_1R_F | CTCAACTCGGGAGAGTCCTG | 2105 (212) |
| 1FL_F | TTGTAGGACGGTGTTGACGG |  |
| 1FL_R | TCCCCGACCCCAACTCTTTA | 1551 (212) |
| Tha_1L_R | CTCGTCCTGCAGTTCATTCA |  |
| **Primers specific for insertion site in 224 line** | | |
| 2FL_F | TTGTGGCATTGATTGCTTTC | 931 (B10) 5534 (224) |
| 2FL_R | CAAAGGCTCCCTCTTCTCCT |  |
| 2FL_F | TTGTGGCATTGATTGCTTTC | 2548 (224) |
| Tha_2L_R | ACAAAAGGGCGACATTCAAC |  |
| Tha_2R_F | CTCAACTCGGGAGAGTCCTG | 1846 (224) |
| 2FL_R | CAAAGGCTCCCTCTTCTCCT |  |
| **Primers specific for insertion site in 225 line** | | |
| 3FL_F | TCTATTAAGGGGTCAGGGGCA | 1469 (B10) |
| 3FL_R | ATCGGCCAACTGCCAACAAT |  |
| 3FL_F | TCTATTAAGGGGTCAGGGGCA | 5895 (225) |
| Tha_3L_R | TGATATTCGGCAAGCAGGCA |  |
| Tha_3R_F | TCGAGAAGGCATCAGCTTGG | 2122 (225) |
| 3FL_R | ATCGGCCAACTGCCAACAAT |  |

**Table S2.** The list of primers used in the verification of predicted polymorphisms.

| **Name** | **Sequence** | **Product length [bp]** |
| --- | --- | --- |
| C138 | CGAGAAGCAAGAATGAGTAGCA | 288 |
| C139 | TTTTGAAAATCATTTCTAATCCAAA |  |
| C140 | GCCGGTAACGATCATTAAACA | 296 |
| C141 | AGTCCACTAGCCCTTGATTTC |  |
| C142 | TTCCTTATTCAAATTATATCGGTTAGA | 352 |
| C143 | AAAGAATTGTGTGCTCATTCCA |  |
| C144 | CATCGCTTTTCTTTGTTTCAA | 400 |
| C145 | TCCAATCATGTGGGTGATCTT |  |
| C146 | TCCAATTTTTCTTCATTTGTGG | 295 |
| C147 | CATAGCACAAACCCTGGCTA |  |
| C148 | TTTTAGAAAACGTGAGAGAGAAAAA | 297 |
| C149 | CGTATGCCTGAATACCTCCAA |  |
| C150 | GGAAACTTCATAGTCATCCTCCA | 300 |
| C151 | TCTTTTCATTACCCGCAAAT |  |
| C152 | GGGTTATCCCTCCATAATGC | 285 |
| C153 | TAATCGGAAATGCCACAACA |  |
| C154 | CGGTGGCTAACTGATAAAGTGA | 400 |
| C155 | TCTTCTTTCATTTTCCGTTTCT |  |
| C156 | CAGAAAACAAATAATGTGGAAACAA | 290 |
| C157 | TTCAGTTGCAGCTTATGCTAGAG |  |
| C158 | GAAGAAAAAGAAATCAGCAGCAA | 292 |
| C159 | GCACCTCGTAATATTCTTTAGGC |  |
| C160 | TGAAGATCCGAAGGAAATGG | 325 |
| C161 | GCCAAAATATGAGCCTAAAGGT |  |
| C162 | TGGGAAGAAACCCAAACAAA | 367 |
| C163 | AAAATGTTCCTTTATTGGTTTATGA |  |
| C164 | CGAAGGTTATGGAACGGGTTA | 343 |
| C165 | TGAAAACCATTTGACACCTCA |  |
| C166 | CGTGAAATTTTCTGATCCTTCA | 379 |
| C167 | GGCCTTAAAAAGTGGGAGCA |  |
| C168 | GAGATCAAGACATTAGAATACGAAAA | 369 |
| C169 | TCAAAATTCTTGGAATTGGATTT |  |
| C170 | CATGGTGAGACGAAAAGTAAATTG | 300 |
| C171 | AACCAATATTTTTGCATCCAAGT |  |
| C172 | TGGAATTCTGCCAGGATGAT | 295 |
| C173 | ACTTGAACATGCAACATAGGC |  |
| C174 | AAAGGCGGTCCTCTTTGAG | 300 |
| C175 | TTCAGCCATCTGTCTTTTGAA |  |
| C176 | GGCAGATGGATAATGGGTTG | 287 |
| C177 | CTCCAACAGTTCGCTGCATA |  |
| C178 | TTTTCAGTAAAGGTTTAATTTTGCTAT | 593 |
| C179 | TTCTCGAGTTTTCAAAATGTACC |  |
| C180 | TTTTTAAAATTCATGCTTGTTTCTTC | 281 |
| C181 | ACAAATTGTTCTGGTTGCTTTT |  |
| C182 | TCGCTTCCCTCAATTCTTTC | 298 |
| C183 | TCTTCCAAACCAGACACGAG |  |
| C184 | AAAAACACCCTAAAATGGACTGA | 268 |
| C185 | TTCCGCTGTGCATGACTTAG |  |
| C186 | CACCCATATGAAACCACAACA | 361 |
| C187 | GCTACCTAAGTCATCAATAAACGAAA |  |
| C188 | TTCAAGTGAGAAGGGCAAAAA | 393 |
| C189 | TTGATGAAAAGGTTCAGCATAAAA |  |
| C190 | GCCTCTCACACACACACACA | 297 |
| C191 | AGGACGTGCAACAAATTGGA |  |
| C192 | TTGTGTTTCCTTTCCAGAATTG | 432 |
| C193 | TTCGGTTAAATCCATTCTCTCA |  |
| C194 | GAGATGTCCATTTCTTTTTCTCTCA | 358 |
| C195 | TCCAAACCATAACTGCGAAA |  |
| C196 | TCATTGATCCCTATCAAACCCTA | 492 |
| C197 | GAGCCTTGGAAGCAACCTTT |  |

**Table S3**. Summary of mapping statistics of three analyzed somaclonal lines.

|  | **212** | **224** | **225** |
| --- | --- | --- | --- |
| **Total number of reads** | 126 867 816 | 126 887 524 | 126 664 932 |
| **Sequencing depth (coverage)** | 34.57× | 34.57× | 34.51× |
| **Total number of filtered reads** | 122 859 014 | 122 741 194 | 123 375 564 |
| **Filtered reads (%)** | 96.84% | 96.73% | 97.40% |
| **Total number of reads mapped** | 105 848 881 | 102 882 010 | 110 413 095 |
| **Total number of reads mapped (%)** | 86.15% | 83.82% | 89.49% |
| **Read mapped uniquely** | 100 674 281 | 97 796 006 | 106 213 817 |
| **Read mapped uniquely (%)** | 95.11% | 95.06% | 96.20% |

**Table S4.** Density of variants predicted in thaumatin lines in reference to the entire genome length.

|  | **Number of SNPs + MNPs** | | **Number of insertions** | | **Number of deletions** | |
| --- | --- | --- | --- | --- | --- | --- |
|  | **In total** | **Per Mb** | **In total** | **Per Mb** | **In total** | **Per Mb** |
| **212** | 1355 | 4 | 344 | 1 | 2847 | 8 |
| **224** | 1170 | 3 | 275 | 1 | 2428 | 7 |
| **225** | 1228 | 3 | 300 | 1 | 2775 | 8 |

**Table S5.** Density of variants counted per 1 Mb of particular regions of the genomic sequence.

|  | Number of variants per 1 Mb of | | | | | |
| --- | --- | --- | --- | --- | --- | --- |
|  | **UTR** | **exons** | **introns** | **upstream** | **downstream** | **intergenic** |
| **212** | 16 | 12 | 20 | 18 | 18 | 37 |
| **224** | 14 | 10 | 18 | 15 | 15 | 31 |
| **225** | 14 | 10 | 17 | 16 | 17 | 36 |
| **average** | 14 | 11 | 18 | 16 | 17 | 34 |

**Table S6.** Percentages of variants having high, moderate, low, or modifier effect on the genome according to SnpEff analysis.

| Effect | **212** | **224** | **225** |
| --- | --- | --- | --- |
| **High** | 1.86% | 1.84% | 1.56% |
| **Moderate** | 0.39% | 0.36% | 0.23% |
| **Low** | 0.43% | 0.38% | 0.32% |
| **Modifier** | 97.32% | 97.42% | 97.89% |

**Table S7**. Number of high-impact (HI) variants and number of genes with HI variants within their structure.

|  | **Total number of HI variants** | **Number of genes with HI variants** | **Number of genes encoding proteins** | **Number of genes with more than one HI variants** |
| --- | --- | --- | --- | --- |
| **212** | 109 | 105 | 105 | 4 |
| **224** | 92 | 89 | 89 | 3 |
| **225** | 87 | 85 | 85 | 2 |


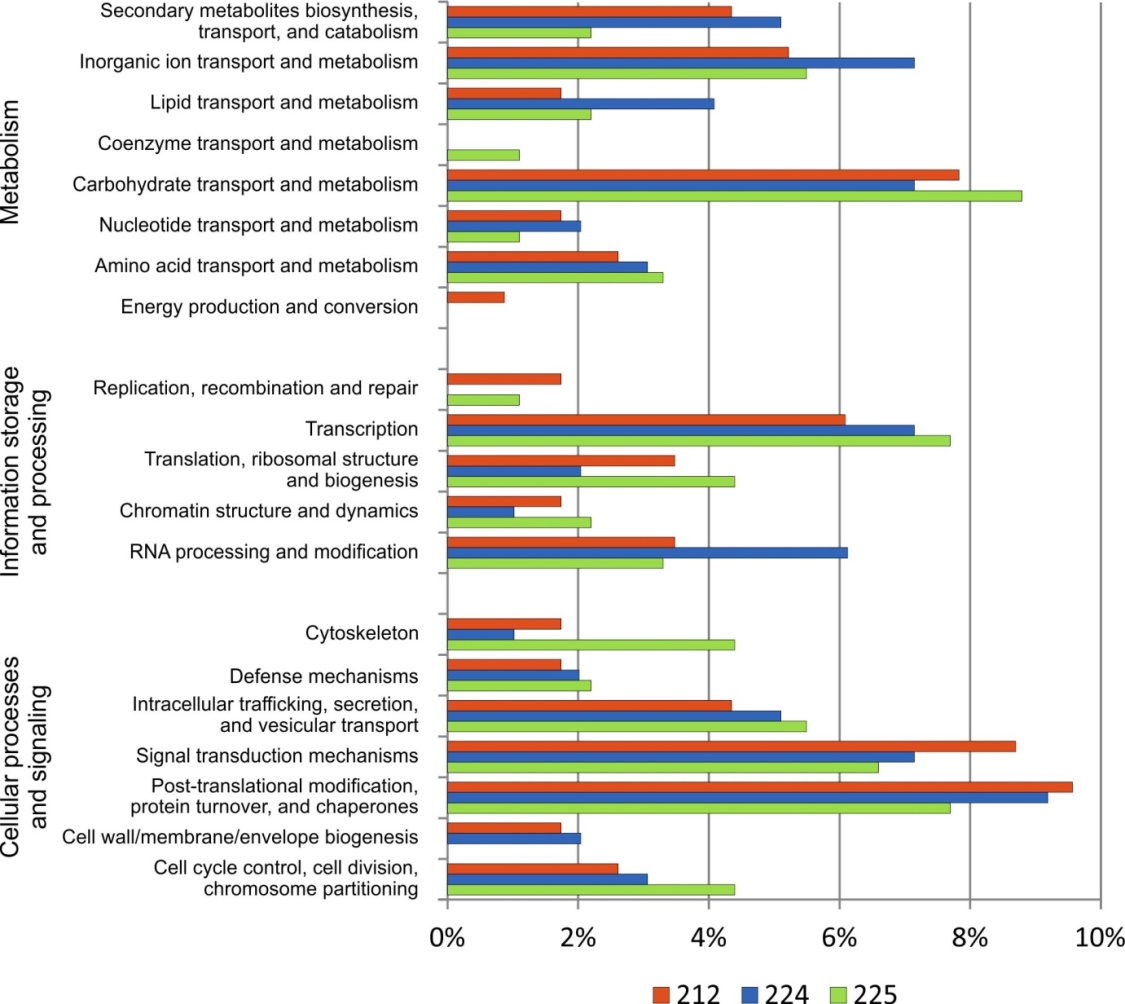


**Figure S1.** Functional KOG classification of genes identified as having HI polymorphisms.

**Table S8**. Number of genes located on ctg1556 with predicted variants inside their structure and number of variants within the genes.

|  |  | **212** | **224** | **225** |
| --- | --- | --- | --- | --- |
| **Number of genes** | with variant in their structure | 35 | 34 | 29 |
|  | with variants in exons | 20 | 21 | 20 |
| **Number of variants** | in genes | 633 | 608 | 557 |
|  | in exons | 65 | 64 | 53 |
